# Transfer of hepatocellular microRNA regulates cytochrome P450 2E1 in renal tubular cells

**DOI:** 10.1016/j.ebiom.2020.103092

**Published:** 2020-11-21

**Authors:** Olivia Matthews, Emma E. Morrison, John D. Tranter, Philip Starkey Lewis, Iqbal S. Toor, Abhishek Srivastava, Rebecca Sargeant, Helen Rollison, Kylie P. Matchett, Timothy J. Kendall, Gillian A. Gray, Chris Goldring, Kevin Park, Laura Denby, Neeraj Dhaun, Matthew A. Bailey, Neil C. Henderson, Dominic Williams, James W. Dear

**Affiliations:** 1 Centre for Cardiovascular Science, The Queen's Medical Research Institute, University of Edinburgh, United Kingdom; 2 Medical Research Council Centre for Regenerative Medicine, University of Edinburgh, United Kingdom; 3 AstraZeneca, Clinical Pharmacology & Safety Sciences Department, Biopharmaceuticals Science Unit, Darwin Building 310, Cambridge Science Park, Milton Rd, Cambridge, CB4 0FZ. United Kingdom; 4 Centre for Inflammation Research, The Queen's Medical Research Institute, University of Edinburgh, United Kingdom; 5 Department of Molecular and Clinical Pharmacology, MRC Centre for Drug Safety Science, University of Liverpool, United Kingdom; 6 MRC Human Genetics Unit, Institute of Genetics and Molecular Medicine, University of Edinburgh, Crewe Road South, Edinburgh EH4 2XU, United Kingdom

**Keywords:** microRNA, Acute liver injury, Kidney function, Signalling, Paracetamol

## Abstract

**Background:**

Extracellular microRNAs enter kidney cells and modify gene expression. We used a Dicer-hepatocyte-specific microRNA conditional-knock-out (Dicer-CKO) mouse to investigate microRNA transfer from liver to kidney.

**Methods:**

Dicer^flox/flox^ mice were treated with a Cre recombinase-expressing adenovirus (AAV8) to selectively inhibit hepatocyte microRNA production (Dicer-CKO). Organ microRNA expression was measured in health and following paracetamol toxicity. The functional consequence of hepatic microRNA transfer was determined by measuring the expression and activity of cytochrome P450 2E1 (target of the hepatocellular miR-122), and by measuring the effect of serum extracellular vesicles (ECVs) on proximal tubular cell injury. In humans with liver injury we measured microRNA expression in urinary ECVs. A murine model of myocardial infarction was used as a non-hepatic model of microRNA release.

**Findings:**

Dicer-CKO mice demonstrated a decrease in kidney miR-122 in the absence of other microRNA changes. During hepatotoxicity, miR-122 increased in kidney tubular cells; this was abolished in Dicer-CKO mice. Depletion of hepatocyte microRNA increased kidney cytochrome P450 2E1 expression and activity. Serum ECVs from mice with hepatotoxicity increased proximal tubular cell miR-122 and prevented cisplatin toxicity. miR-122 increased in urinary ECVs during human hepatotoxicity. Transfer of microRNA was not restricted to liver injury –miR-499 was released following cardiac injury and correlated with an increase in the kidney.

**Interpretation:**

Physiological transfer of functional microRNA to the kidney is increased by liver injury and this signalling represents a new paradigm for understanding the relationship between liver injury and renal function.

**Funding:**

Kidney Research UK, Medical Research Scotland, Medical Research Council.

**Research in context**

**Evidence before the study**

The commonest cause of acute liver injury is paracetamol (acetaminophen) overdose. When the liver is injured, renal function is crucial in determining patient outcome. There is limited understanding of the pathways that regulate the renal response to liver injury. Such pathways may represent potential drug targets. It is established that a microRNA species, miR-122, is highly expressed specifically in hepatocytes. It is released into the circulation in high concentration following paracetamol-induced liver injury in human disease and murine models. MicroRNAs can enter recipient cells to modulate their RNA targets. We tested the hypothesis that functional miR-122 is transferred from the liver to the kidney following liver injury.

**Added value of this study**

When liver microRNA was depleted in a mouse model there was a fall in miR-122 (hepatocyte-enriched) in the kidney. Conversely, following paracetamol-induced liver injury there was increased liver-derived miR-122 in kidney tubular cells. Liver-derived microRNA regulated kidney cytochrome P450 2E1 and inhibited toxic injury. This signalling pathway is potentially active in humans – urinary miR-122 was increased with liver injury.

**Implications of all available evidence**

miR-122 is released from the injured liver and transferred to the kidney where it has the potential to modulate the renal response to injury. This microRNA already represents a drug target - miravirsen, a locked nucleic acid-modified DNA antisense oligonucleotide which specifically binds miR-122 - has demonstrated efficacy in a clinical trial to treat hepatitis C. With development, medicines which target miR-122 may also be effective treatments to prevent renal failure in patients with liver disease.

Alt-text: Unlabelled box

## 1 Introduction

MicroRNAs are key regulators of gene expression [1]. The primary microRNA transcript is cleaved in the cell nucleus to release a pre-microRNA that is exported into the cytoplasm. The cytoplasmic enzyme Dicer cleaves the pre-microRNA to produce a microRNA duplex, one strand of which is loaded onto an Argonaute protein to form the RNA silencing complex [1]. In the circulation, cell-free microRNAs are protected from degradation by extra-cellular vesicles (ECVs) and protein complexes such as Argonaute [2], and they are potential disease biomarkers [3]. Extra-cellular microRNAs can enter cells and change gene expression and cellular function *in vitro* [4]. There is evidence that transfer of microRNA between organs occurs *in vivo* in pre-clinical models of diseases such as cancer [5]. Therefore, extra-cellular microRNAs may represent a new class of signalling molecules and drug targets. In this paper, we tested the hypothesis that microRNAs produced in the liver are transferred to regulate gene expression in the kidney. We focussed on the kidney because our earlier work demonstrated that microRNA species can enter kidney tubular cells under physiological hormonal control and modulate their mRNA targets [6]. Also, there is a clinically important relationship between acute liver injury and kidney injury. For example, kidney function forms an integral part of all the clinical risk stratification models that are used to make decisions regarding need for liver transplantation to prevent death in patients with acute liver failure [7].

MicroRNA-122 (miR-122-5p, miR-122) is highly expressed in hepatocytes (∼40,000 copies per cell – around 70% of total hepatocyte microRNA) [8,9]. In patients with acute liver injury, and in pre-clinical models of liver injury such as paracetamol toxicity, the circulating concentration of miR-122 is increased 100–1000 fold [10]. In over 1000 patients we have demonstrated that miR-122 is a sensitive and specific biomarker of acute liver injury risk after paracetamol (acetaminophen) overdose, the most common cause of acute liver failure in the Western world [11].

The cytochrome P450 (CYP) enzymes, particularly CYP2E1, are responsible for generating the toxic metabolite (*N*-acetyl-*p*-benzoquinone imine - NAPQI) that causes cell death in the context of paracetamol toxicity [12]. In the liver, CYP2E1 is established as being regulated by miR-122 [13,14]. In the kidney, CYP2E1 is expressed in tubular cells, a cellular location where it can also mediate drug toxicity. For example, although rare, paracetamol is well recognised to directly produce acute kidney injury in the absence of liver injury [15]. CYP2E1 deletion in mice prevents cisplatin-induced acute kidney injury demonstrating a central role for CYP2E1 in this well-established model of nephrotoxicity [16]. In this paper, we explore whether liver to kidney microRNA transfer regulates kidney CYP2E1 expression and activity, and whether liver-derived microRNA can modulate nephrotoxic tubular cell injury.

## 2 Methods

### 2.1 Animal Studies

Mice with free access to standard chow and water were housed in groups of 4–8 in open-top cages, at 22 °C ± 1 °C, 55% humidity and on a 12 h light dark cycle (lights on at 07:00). The mice were allowed to acclimatise to the environment for at least a week prior the start of each experiment. At the end of the study, mice were euthanized by rising CO_2_ exposure followed by exsanguination, unless stated otherwise.

#### 2.1.1 Dicer conditional knock-out (Dicer-CKO)

Dicer^flox/flox^ mice (male and female, 2-3 months old) (B6.Cg-*Dicer1^tm1Bdh^*/J strain) were injected with a single tail vein injection of the hepatocyte-specific AAV8.TBG.PI.Cre.rBG (AAV8-Cre) or AAV8.TBG.PI.Null.bGH (AAV8-null) (Penn Vector Core, Pennsylvania, USA, 2.5 × 10^11^- 6.25 × 10^10^ viral genomes/100 µl dose in sterile PBS). Untreated mice Dicer^flox/flox^ (baseline) were used as controls where indicated. After 1–4 weeks, blood and tissue were collected. Whole blood was spun for 10 min at 8000 x *g*, 4 °C, serum was isolated and stored at -80 °C. Tissue was placed in 4% paraformaldehyde (Sigma Aldrich) for 24 h, followed by storage in 70% ethanol and finally paraffin embedded for sectioning. The rest of the tissue was put into RNAlater (Sigma Aldrich) and snap frozen for long-term storage at -80 °C. Prior to tissue collection the circulation was perfused with sterile saline. Where indicated in the results section, mice were treated with paracetamol as described below.

#### 2.1.2 Paracetamol hepatotoxicity model

Wild-type male C57BL/6JCrl mice (2–3 months old) or Dicer-CKO mice were fasted overnight for 12 h then intraperitoneally injected with paracetamol (ACROS organics, Geel, Belgium) dissolved in sterile PBS (Sigma Aldrich, Dorset, UK). Tissue and blood collection occurred 6 hours after paracetamol injection. This time was chosen as it is the peak time for circulating miR-122 after liver injury. Prior to tissue collection the circulation was perfused with sterile saline. Immediately following tissue collection, tissue RNA integrity was stabilised and protected from degradation by collection into RNA*later* and immediate storage at -80 °C. RNA quantity and purity were assessed using the NanoDrop® ND-1000 UV-Vis Spectrophotometer (Thermo Fisher Scientific) prior to miRNA and mRNA analysis. In specific experiments, circulatory ECVs were isolated from mouse serum as described previously [17]. In brief, mouse serum was vigorously vortexed then centrifuged at 12,500 x *g* for 30 min. The supernatant was then centrifuged at 120,000 x *g* for 70 min to pellet the ECV fraction. The pellet was washed and then re- centrifuged before final resuspension in PBS. In certain studies pelleted ECVs were conjugated with Cell Tracker 655 (Invitrogen, CA, USA) following the manufacturer's protocol. ECV size distribution and number was measured by nanoparticle tracking analysis as previously described [18]. Mouse plasma alanine transaminase activity (ALT) was determined using a commercial serum ALT kit (Alpha Laboratories Ltd., Eastleigh, UK) adapted for use on either a Cobas Fara or Cobas Mira analyser (Roche Diagnostics Ltd, Welwyn Garden City, UK). In certain studies, kidney cells were isolated by fluorescence-activated cell sorting (FACS). Kidneys were dissociated in RPMI media containing collagenase II (Sigma-Aldrich C6885), collagenase D (Roche 11088858001), dispase (Gibco 17105041) and DNase I (Roche 04716728001). Cells were then incubated with BD Fc Block™ purified anti-mouse CD16/CD32 (BD Biosciences 553141) prior to incubation with fluorescein-labelled *lotus tetragonolobus lectin* (LTL, Vector Laboratories FL-1321), Brilliant Violet 605™ anti-mouse CD31 (Biolegend 102427) and APC rat anti-mouse CD45 (BD Bioscience 561018). FACS was performed on the BD FACSAria™ II apparatus (BD Biosciences).

#### 2.1.3 Coronary artery ligation (CAL) model

CAL was performed in wild-type C57/BL6 mice as described previously [19,20]. In brief, an incision was made in the lower thorax and the chest opened at the fourth intercostal space and held open using retractors. The left anterior descending coronary artery was ligated in mice randomised to the CAL group, at the level below the left atrium. After ligation, the chest was closed. For mice undergoing sham surgery, thoracotomy alone was performed; ligation of the left anterior descending coronary artery was not conducted. An ultra-sensitive mouse cardiac troponin-I ELISA (Life Diagnostics, Stoke on Trent, UK) was used on serum as per the manufacturer's instructions.

### 2.2 Human studies

Adult patients (age>16 years) admitted to the Royal Infirmary of Edinburgh, UK following paracetamol overdose without or with acute liver injury (ALT>1000U/L) were included. Urine was collected and ECVs were isolated from the whole urine samples as previously described [18].

### 2.3 In vitro studies

#### 2.3.1 Primary proximal tubular cell isolation

Male mice were euthanised and kidneys were removed immediately under aseptic conditions and the cortex macroscopically dissected. The cortex was minced and incubated with 1 mg/ml collagenase I and IV (Sigma Aldrich, Dorset, UK) for 45 min at 37 °C. The resultant solution was ground and serially sieved to a final filter size of 40 µm. The filtered solution was centrifuged at 27,000 x *g* through a 48% Percoll gradient (Sigma Aldrich, Dorset, UK) and each of the 4 distinct bands were carefully removed (F1-4). Cells contained within the lowermost band (F4) were washed and passed through a 40 µm sieve. The cells were resuspended in DMEM/Hams's F12 media with glutamax containing: 5 μg/ml insulin, 50 nM hydrocortisone, 10 ng/ml EGF, 5 μg/ml transferrin, 50 nM sodium selenite, 10 nM triiodothyronine, 100 U/ml penicillin, 100 μg/ml streptomycin and 1% (wt/vol) exosome depleted FBS (System Biosciences, CA, US). Studies of NHE3 and KIM-1 protein expression and alkaline phosphatase activity were used to confirm the isolation of proximal tubular cells (PPT). PPT cells were seeded on at a cell density of 5 × 103 cells/well for 48 hours. Then circulatory ECVs were co-incubated with cells at a concentration of 250 × 10^8^/ml media, 48 h prior to induction of injury. Nephrotoxic injury was induced by the addition of cisplatin (Cambridge Bioscience) to PPT cells for 48 h. Experiments were conducted in technical triplicate (3 wells) and biological quintuplicate (ECVs isolated from 5 animals per group). Cell viability was determined by the CellTiter-Glo® cell viability assay (ATP quantification) (Promega, Wisconsin, USA) assay and the CellTiter 96® Aqueous Assay (MTS assay) (Promega, Wisconsin, USA) as per manufacturer's instructions.

#### 2.3.2 RNA preparation and qRT-PCR

Total RNA was extracted and purified from serum using the miRNeasy Serum/Plasma Kit (Qiagen) with *C.elegans* miR-39 (Qiagen) added as an external control. Tissue total RNA (250 ng) was extracted and purified using the miRNeasy Mini Kit (Qiagen). cDNA was synthesised using the miScript II RT kit (Qiagen) according to the manufacturer instructions and diluted 1:10 to perform qRT-PCR. MicroRNA and mRNA quantification was carried out using the miScript SYBR green PCR kit (Qiagen) on the Lightcycler 480 (Roche Diagnosis). Quantitect and miScript primers were purchased from Qiagen. For the quantification of the primary miR-122 transcript, the Taqman Gene Expression Mix was used for the detection of primary miR-122 transcripts (Life Technologies).

#### 2.3.3 Histological scoring (necrosis quantification)

Liver H&E sections were scored for injury as follows: 100x (distance (µm) between the central vein and edge of the necrotic zone)/ (total distance (µm) between the central vein and portal triad). 10 measurements chosen at random were made per section.

#### 2.3.4 Immunohistochemistry

CYP2E1 immunohistochemistry in the liver and kidney was performed on the Discovery ULTRA Staining Module (Roche Diagnostics). For staining preparation, the sections were exposed to 100 °C (4 min) and incubated with Ventana Cell Conditioner 1 (5 × 8 min). Ventana DISCOVERY inhibitor (8mins), casesin (1:10,8 min) were then placed on the slides. With the slides warmed up to 37 °C, the Cytochrome P450 2E1 antibody (1:100, Abcam, Ab28146) (60mins) and casesin (1:10, 1 × 8 min) were used. Next, the slides were exposed to anti-rabbit IgG H (Roche DISCOVERY UltraMap, 760–4315) (10mins). To initiate the signal, the HRP-activated chromagen, DISCOVERY purple was added (40 min). Finally, Haemotoxylin, followed by bluing reagent was used as the counterstain.

#### 2.3.5 Western blot

CYP2E1 and Dicer protein expression was quantified by western blot analysis. Protein homogenate was denatured at 70 °C for 15 min and each sample was added at 30 µg to either 4–12% or 4–20 % tris-glycine precast gels (Novex™, Wedgewell™, Invitrogen). Following 1 h of blocking with 5% milk (diluted in TSB-T wash buffer), the primary antibodies **(Supplementary Table 1**) were added to 1% milk (dissolved in TBS-T) and incubated with the membranes (overnight, 4 °C). The membranes were washed with TBS-T (3 × 10 min) and then transferred into the HRP-secondary antibody **(Supplementary Table 1)** (diluted in 1% milk, 1 hour, at room temperature). The membranes were washed as previously described and incubated with ECL reagent (Pierce™, ThermoFisher Scientific) (5 min, at room temperature). The membranes were exposed to X-Ray film (CL-Xposure film, ThermoFisher Scientific), developed (Compact 4x, Xograph) and scanned. After the film development, the membranes were incubated in acid stripping buffer (2 × 30 min) and washed with PBS (3 × 3 min) ready to re-probe for β-actin following the methods above.

#### 2.3.6 In situ hybridisation

miRNA-122 was localised in the liver using the Discovery ULTRA Staining Module (Roche Diagnostics). During the staining programme, the miRCURY LNA miRNA Detection probes (mmu-miR-122-5p, YD00615338) and controls (U6, YD00699002 and Scramble-miR, YD00699004) were added. These were prepared using the miRCURY LNA miRNA ISH Buffer Set (FFPE) (Qiagen) mixed 1:1 with UltraPure DEPC water (Thermo Fisher Scientific).

#### 2.3.7 Cytochrome p450 2E1 activity measurements

Tissue was homogenised into a microsomal preparation. The liver and kidney protein was diluted to 1 mg/ml and 3 mg/ml, respectively in phosphate buffer (pH 7.4). To determine the CYP2E1 metabolism activity, the sample was added to the CYP2E1 substrate, chlorzoxazone and NADPH mixture. The reaction was halted by a quench solution containing 200 nM of benzoxazol and 4 nM of verapamil 10 min after the incubation. Analyte measurements were made on the Xevo Water TQ-S Mass Spectrometry machine using the Water's Masslynx software (Waters Corporation, Massachusetts, United States). Response of the CYP2E1 enzyme was determined from: $\frac{\text{AUC of analyte}}{\text{AUC of internal control (Verapamil)}}$

### 2.4 Statistical analysis

All data are presented as median±IQR. When two groups were compared the Mann-Whitney Test was used. For time course data on separate mice per time point linear regression was performed then each treatment was compared to null treatment to determine whether slopes were significantly different.

### 2.5 Study approval

All animal studies were performed in accordance with the Animals (Scientific Procedures) Act 1986 / ASPA Amendment Regulations 2012 following ethical review by the University of Edinburgh. The local research ethics committee prospectively approved the human study, and informed consent was obtained from all patients before entry into the study.

### 2.6 Role of funders

The funders of the study had no role in study design, data collection, data analysis, data interpretation, or writing of the report. The corresponding author had full access to all the data in the study and had final responsibility for the decision to submit for publication.

## 3 Results

### 3.1 Basal miR-122 in the kidney originates from the liver

**Fig. 1:**
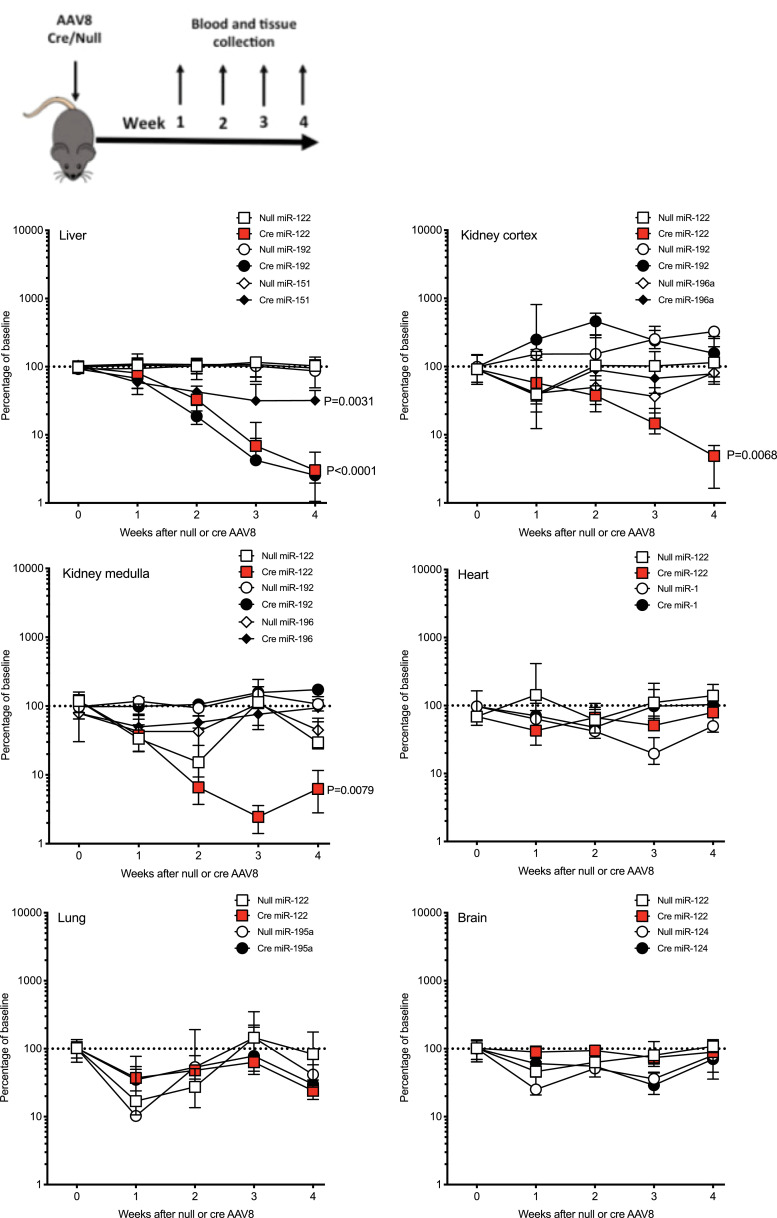
The percentage changes over time in organ microRNA expression after treatment of Dicer^flox/flox^ mice with AAV8 vector expressing or not expressing Cre Recombinase (Cre). Schematic for study design is presented. In graphs, for each organ, the expression of microRNAs is expressed as a percentage of the untreated Dicer^flox/flox^ mice (baseline). Data are normalised to U6. Filled symbols represent mice receiving AAV8-Cre. Unfilled symbols represent AAV8-null. Symbols represent the time point median and the error bars define the inter-quartile range. N=20 for each AVV8/microRNA combination (N=5 for each time point). Linear regression was performed then for each microRNA Cre treatment was compared to null treatment to determine whether slopes were significantly different.

To determine whether liver hepatocellular microRNA is transferred to other organs, we depleted hepatocyte microRNA by treating Dicer^flox/flox^ mice with a Cre recombinase-expressing adenovirus (AAV8-Cre) (or control AAV8 which did not express Cre (AAV8-null))**.** AAV8 treatment did not result in histological liver injury but modestly increased serum ALT activity. There was no difference between Cre and null. Liver histology was reviewed by a pathologist blinded to the treatment grouping (author TJK). Portal-central vascular relationships were normal. Portal tracts were uninflamed, and there was no bile duct inflammation, damage or loss. There was no interface inflammation or ductular reaction. No lobular inflammation, hepatocellular injury or loss. No steatosis **(Figure S1).** Exposure to AAV8-Cre resulted in a time-dependent decrease in liver Dicer expression at the mRNA and protein level **(Figure S2 and S3).** AAV8-null had no effect on Dicer. Liver depletion of Dicer resulted in a time-dependent decrease in miR-122, miR-192 and miR-151 (microRNA species enriched in the liver [21]) **(**Fig. 1 **and S4).** There was no significant microRNA change following AAV8-null injection. To complement our PCR data and confirm miR-122 depletion, we performed in situ hybridisation (ISH) on liver tissue, which demonstrated reduced tissue expression with AAV8-Cre treatment **(Figure S5)**. In serum, miR-122 concentration was reduced when AAV-Cre treated mice were compared with AAV8-null (normalised to miR-39. Week 3 after AAV8 injection: AAV8-null median 7.4 (IQR 3.3–54.7); AAV8-Cre 1.3 (0.2–3.1). Week 4 after AAV8 injection: AAV8-null 4.8 (IQR 0.3-88.1); AAV8-Cre 0.3 (0.1–1.5) *N*=5 per group).

No change in Dicer expression in the kidney (cortex and medulla), heart or lung occurred, highlighting the liver specificity of Cre delivery by the AAV8 vector **(Figure S2).** There was a decrease in brain Dicer following AAV8-Cre. miR-122 concentration in these non-hepatic organs was determined alongside selected, organ-enriched, microRNAs (kidney: miR-192, miR-196a; heart: miR-1; lung: miR-195a; brain: miR-124) [9]. The only microRNA to significantly decrease following AAV8-Cre treatment of mice was miR-122 in the kidney cortex and medulla **(**Fig. 1 **and S4).** To determine whether miR-122 was transcribed in the kidney we measured the primary miR-122 transcript. It was barely detectable in the kidney cortex and medulla (expression normalised to *Gapdh* – kidney cortex median 0.0023 (IQR 0.0016–0.003), in contrast to liver pri-miR-122 1.7 (0.9–5.4) *N*=5 per group) and was unaffected by AAV8 treatment (3 weeks after AAV8: null 0.0035 (0.0028–0.0044). Cre 0.0037 (0.0026–0.005) *N*=5 per group). Therefore, the change in miR-122 in the kidney was not associated with a change in *de novo* synthesis in the kidney cells.

### 3.2 Increased miR-122 transfer to the kidney occurs following liver injury in mice and humans

**Fig. 2:**
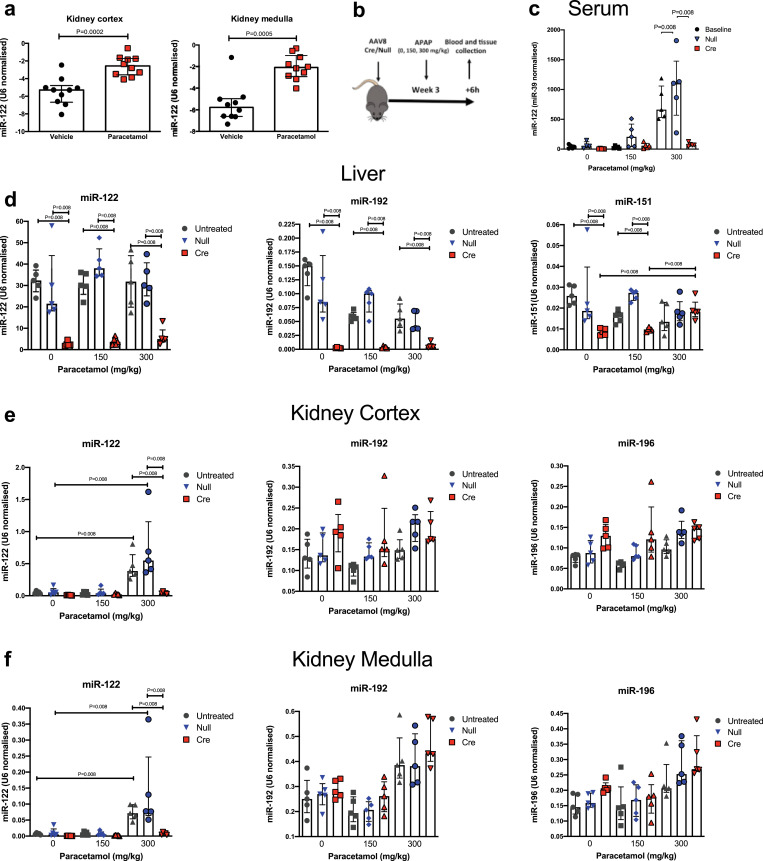
a) miR-122 was increased in the kidney cortex and medulla 6 hours after mice were treated with paracetamol (300mg/kg i.p.). N=10 per group. b) Schematic overview of study (APAP = paracetamol). C-F) Dicer^flox/flox^ mice were treated with AAV8 vector expressing or not expressing Cre recombinase (Cre or null). 3 weeks after AAV8 treatment mice received paracetamol 150 or 300 mg/kg (or vehicle (0)) ip, then serum (c), liver (d), kidney cortex (e) and kidney medulla (f) were harvested 6 hours later. Untreated = Dicer^flox/flox^ mice not receiving AAV8. MicroRNA expression is expressed as U6(ct) - miR (ct). N=5 per group. Statistical significance was determined by Mann-Whitney Test. Data are represented individual mice with bars representing median and IQR.

We treated wild-type (C57BL/6) mice with a hepatotoxic dose of paracetamol (300 mg/kg). Published studies demonstrate substantially increased circulating miR-122 6 h after dosing, so this time window was chosen to study transfer to the kidney [22]. In our study, after 6 hours, paracetamol resulted in centri-lobular hepatocyte necrosis and an elevation in serum ALT activity (300 mg/kg dose, around 38 fold) and serum miR-122 concentration (around 18-fold). Interestingly, hepatic expression of miR-122 was significantly altered after paracetamol exposure. There was loss of miR-122 from necrotic centri-lobular hepatocytes but a marked increase in expression in the areas surrounding the necrotic cells with the highest expression in the viable hepatocytes closest to the injured areas **(Figure S5).** Consistent with hepato-renal transfer, the concentration of miR-122 increased in the kidney cortex and medulla **(**Fig. 2**).** To confirm that the liver was the source of the increase in miR-122 in the kidney we repeated the experiment with Dicer deletion using the AAV8Cre/Null (or no AAV8) followed 3 weeks later by paracetamol exposure (0, 150 and 300 mg/kg). In this model paracetamol treatment resulted in histological and biochemical liver injury with higher ALT activity and increased necrosis in the AAV8-Cre treated group **(Figure S6)**. After AAV8-Cre treatment, miR-122 expression in the liver tissue following paracetamol exposure was lost in the areas surrounding necrotic cells **(Figure S5).** As in the wild-type mice, there was a significant increase in kidney cortex and medulla miR-122 in untreated and AAV8-null treated Dicer^flox/flox^ mice. This increase was substantially attenuated when the Dicer^flox/flox^ mice were treated with the AAV8-Cre vector (14-fold difference when AAV8-Cre compared to AAV8-null after 300 mg/kg paracetamol) **(**Fig. 2**).** There was no effect on Dicer in the kidney **(Figure S7)** and no change in miR-192 or miR-196a **(**Fig. 2**)**. There was no expression of the primary miR-122 transcript in the kidney cortex and medulla without or with paracetamol treatment in any treatment group (Ct value >35). To determine which kidney cell type had increased miR-122 after liver injury, we FACS sorted kidney cells. There was a significant increase only in the miR-122 content of LTL+ tubular cells **(Figure S8).**

**Fig. 3:**
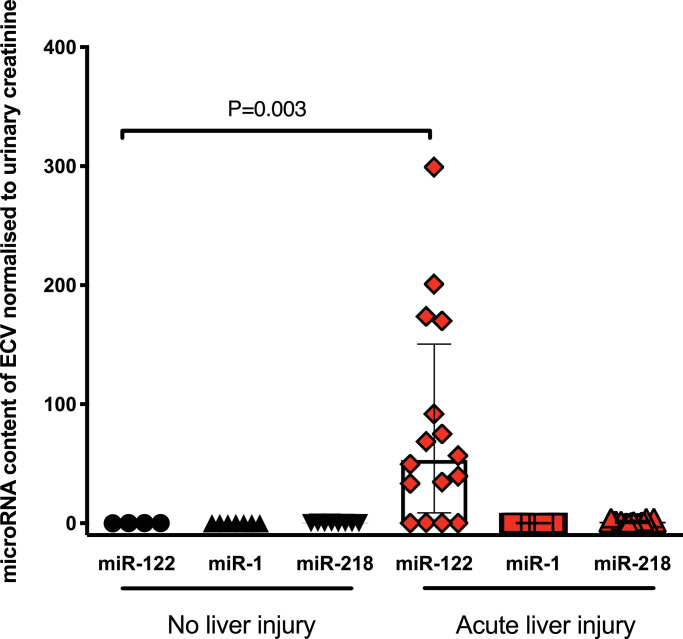
Urinary extra-cellular vesicles (ECVs) contain miR-122, which was significantly increased in patients with paracetamol-induced acute liver injury. Urine was collected from patients who had taken a paracetamol overdose that required treatment but did not cause liver injury ('No liver injury') and patients with liver injury (ALT>1000U/L). ECVs were isolated by ultra-centrifugation. MicroRNA concentration was measured and expression normalised by urinary creatinine. Statistical significance was determined by Mann-Whitney Test. Data are represented individual patients with bars representing median and IQR (N=4-20 per group).

To begin to translate this pre-clinical work into humans, we measured miR-122 in urinary ECVs from patients following paracetamol overdose (without and with acute liver injury, serum ALT activity >1000U/L – **Supplementary Table 2**). miR-122 was substantially increased in the urinary ECVs of those patients with liver injury demonstrating transfer of miR-122 from circulation to the urinary space in humans with paracetamol hepatotoxicity **(**Fig. 3**).** There was no change in two microRNAs enriched in non-hepatic organs.

### 3.3 Cardiac microRNA is transferred to the kidney following myocardial ischaemia

To determine whether microRNA transfer to the kidney is restricted to liver injury we explored kidney microRNA expression in a model of ischaemic cardiac injury induced by coronary artery ligation (CAL). Cardiac injury was demonstrated by an increased circulating troponin concentration 6 hours after CAL (Sham: 1.1 ng/mL (0.9-1.9). CAL: 22.4ng/mL (17.0–24.5) *P*=0.016 *N*=5 Mann-Whitney Test). A panel of microRNAs were measured in the circulation and miR-499 was identified as the species with the largest fold increase with CAL (relative fold increase from baseline 159 (116-813), data not shown for other species). This increase in the circulation was associated with a significant decrease in the expression of miR-499 in the left ventricle of the heart and a concurrent increase in the kidney **(Figure S9).** This demonstrates that microRNA released from the heart could also be transferred to the kidney.

### 3.4 Kidney CYP2E1 and toxic injury is regulated by liver-derived microRNA

**Fig. 4:**
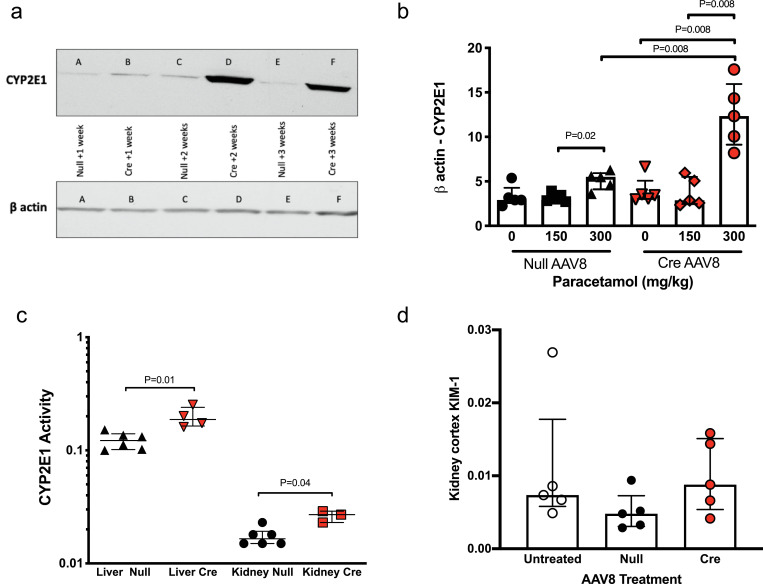
Dicer^flox/flox^ mice were treated with AAV8 vector expressing or not expressing Cre recombinase (Cre or null). a) Western blot of kidney cortex CYP2E1 A, C, E. mice treated with AAV8 null then kidney collected 1, 2 or 3 weeks later. B, D, F mice treated with AAV8 Cre then kidney collected 1, 2 or 3 weeks later b) Cytochrome P450 2E1 (CYP2E1) mRNA expression is expressed as b-actin(ct) - CYP2E1 (ct). 3 weeks after AAV8 treatment mice received paracetamol 150 or 300 mg/kg (or vehicle (0)) ip, then the kidney cortex was harvested 6 hours later. c). CYP2E1 activity was determined by metabolism of chlorzoxazone.d) Kidney injury molecule 1 (KIM-1) mRNA expression in the kidney cortex following paracetamol (300mg/kg ip) in no AAV8 treated (untreated), AAV8 null and AAV8 Cre treated mice. KIM-1 is normalized by beta-actin. For a-d, statistical significance was determined by Mann-Whitney Test. Data are represented individual mice with bars representing median and IQR (N=5 per group).

In Dicer ^flox/flox^ mice pre-treated with AAV8-Cre, kidney CYP2E1 was significantly increased at the protein and RNA level compared to AAV8-null in untreated mice and after paracetamol exposure **(**Fig. 4 **a&b, S10).** To confirm that enzyme activity is regulated by liver microRNA we used the chlorzoxazone probe to assay CYP2E1 activity in liver-derived microsomes. There was a significant increase in liver CYP2E1 enzyme activity in Dicer ^flox/flox^ mice treated with AAV8-Cre compared to AAV8-null treatment **(**Fig. 4**c).**Furthermore, there was increased CYP2E1 activity in kidney-derived microsomes from AAV8-Cre treated mice **(**Fig. 4**c)**. CYP2E1 is the main enzyme responsible for producing the toxic metabolite of paracetamol. Therefore, we investigated whether there was evidence of renal injury in the AAV8-Cre treated mice (with lower miR-122 and higher CYP2E1 in the kidney). There was no histological evidence of injury (data not shown). At the molecular level, we measured kidney injury molecule 1 (KIM-1) mRNA expression (an FDA approved biomarker of proximal tubular injury) [23]. There was no difference in KIM-1 expression when AAV8 treatment groups were compared **(**Fig. 4**d).**

**Fig. 5:**
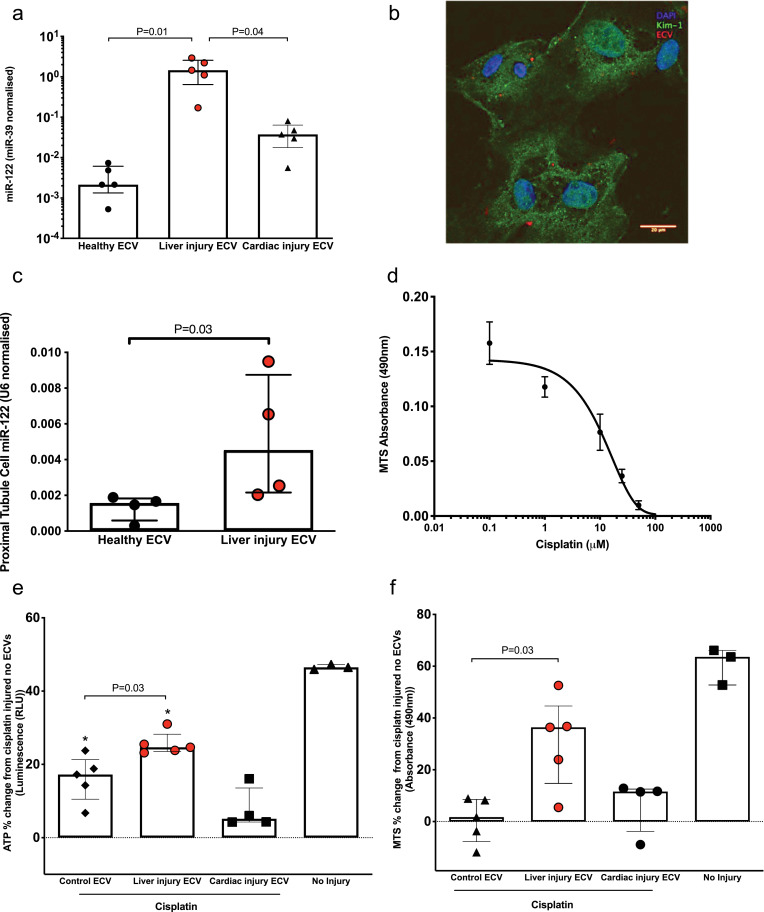
a) miR-122 in ECVs from serum of healthy mice, mice with liver injury (300mg/kg paracetamol IP) or cardiac injury (induced by CAL). b). Murine primary proximal tubular (PTT) cells internalize ECVs (red). Green represents KIM-1, an archetypal proximal tubular protein. Blue stain is DAPI. c) ECVs were isolated from the serum of mice treated with a toxic dose of paracetamol (300mg/kg ip - liver injury ECV) or healthy mice. Equal ECV numbers were applied to PTT cells and the miR-122 content of the cells was determined. d) PPT cells were exposed to cisplatin for 48 hours prior to assessment of cell viability by NADPH activity (N=4). e & f). PTT cells were co-incubated with circulatory ECVs (250 × 10^8^/ml) for 48 hours and subsequently injured with 10 μM cisplatin. Circulatory ECVs were derived from healthy mice (control), liver injury mice (300mg/kg paracetamol IP) or cardiac injury induced by CAL. Cells that were not injured with cisplatin and had no additional ECVs added are labeled no injury. All results were normalised to the results from PTT cells injured with 10 μM cisplatin with no additional ECVs (baseline). Data represents ATP concentration (Figure e) and NADPH activity (Figure f) as percentage change from baseline. Statistical significance was determined by Mann-Whitney Test. Data are represented individual mice with bars representing median and IQR (N=5 per group). *=P=0.008 compared to baseline.

Paracetamol does not consistently produce kidney injury in pre-clinical models or humans following overdose [24]. Therefore, we developed an *ex vivo* model to determine whether circulating microRNA released from the injured liver can reduce nephrotoxicity. We have previously demonstrated that kidney tubular epithelial cells can take up ECVs and these can deliver functional microRNA into the cells [6]. In serum from healthy mice we separated ECVs from the non-ECV fraction. Utilising miR-122 copy number and calculating the average copies of miR-122 in both fractions quantified the relative distribution of miR-122. The majority of miR-122 (79.8±6.9%) was located in the ECV fraction. Therefore, we hypothesised that ECVs transfer miR-122 from liver to kidney. ECVs were isolated from mouse serum and their size distribution was characterized by nanoparticle tracking analysis. The miR-122 concentration of the ECVs from mice with liver injury due to paracetamol was around 460 and 39 fold higher than untreated and cardiac injury ECVs, respectively **(**Fig. 5**a)**. Mouse primary proximal tubular cells were isolated **(Figure S11).** Fluorescently-labelled ECVs from liver injury mice entered mouse primary proximal tubular cells **(**Fig. 5**b)** and resulted in a significant increase in proximal tubular cell miR-122 concentration **(**Fig. 5**c)**. To determine whether microRNA release from the liver could prevent drug toxicity in the kidney we cultured mouse primary proximal tubular cells with equal numbers of ECVs isolated from the following groups: circulation of untreated mice, mice with liver injury induced by paracetamol or mice with cardiac injury induced by CAL. ECV treatment for 48 h was followed by exposure of the cells to cisplatin (the toxicity of which is dependent on CYP2E1 activity [16]) at the EC50 toxicity dose **(**Fig. 5**d)**. The ECVs from mice with liver injury significantly attenuated cisplatin toxicity. By contrast, ECVs from healthy mice and mice with cardiac injury had no effect **(**Fig. 5**e&f).**

## 4 Discussion

The work described in this paper demonstrates that miR-122 is transferred from the liver to the kidney tubular cells as a physiological process (in the uninjured mouse) that is increased by acute liver injury. In the kidney, microRNA originating from the liver regulated CYP2E1 RNA and protein expression and activity and attenuated cisplatin toxicity. In humans with paracetamol toxicity there is evidence of increased miR-122 in urinary ECVs, which supports our mouse data being a faithful reflection of human pathophysiology. miR-122 has two unique properties which facilitated our experiments. Firstly, it is highly enriched in hepatocytes compared to other organs. Data from the FANTOM consortium demonstrates that miR-122 is approximately 18,000 higher in hepatocytes than renal proximal tubular cells [9]. Secondly, it is released in large amounts from the injured liver into the circulation. These properties allowed us to use miR-122 to track endogenous microRNA transfer by liver-specific deletion of the enzyme that generates mature microRNA species (Dicer). This approach also allowed us to confidently exclude off-target Cre recombinase activity in non-hepatic organs by measurement of Dicer and organ-selective microRNAs. When Dicer was deleted in the liver there was a subsequent substantial miR-122 decrease in the kidney. The primary transcript for miR-122 was undetectable in the kidney. The FANTOM consortium used RNA-sequencing and cap analysis of gene expression (CAGE) across multiple primary cell types. This demonstrated the presence of mature miR-122 in kidney proximal tubular cells without expression of its promoter [9]. This supports the conclusion from our studies - miR-122 expression in the kidney results from transfer from the liver. This is consistent with the results of Rivkin *et al.* who used macrophage depletion or antagomirs to indirectly inhibit LPS-induced miR-122 release from mouse hepatocytes and reported a corresponding reduction of kidney miR-122 [25]. The data presented in our paper now demonstrate that microRNA transfer from the liver to the kidney occurs as part of normal physiology and does not require experimental induction of inflammation or tissue injury. It is likely that multiple microRNA species are transferred from liver to kidney as part of normal physiology. The unique properties of miR-122 described above allow us to detect a change whereas other microRNAs will be synthesised locally in the kidney. This local expression is likely to mask any change due to decreased transfer from the liver (a hypothesis supported by miR-192 not changing in the kidney despite a decrease in the liver). Future studies should define the total contribution of liver-derived RNA in the kidney.

Circulating microRNAs change with tissue injury as exemplified by the increase in miR-122 that accompanies liver injury. In this paper we demonstrate that miR-122 released as a result of paracetamol toxicity targets the kidney tubular cells. miR-122 did not increase in the kidney of the null mice (without paracetamol treatment) despite some of the mice having an increase in ALT activity (supplementary figure 1). We speculate that this reflects miR-122 circulating concentrations being lower after AAV8 exposure than following paracetamol (3 weeks after AAV8 null median (IQR) 3523 (1056–37,548); 4 weeks 2086 (79–53,734); post-paracetamol 300 mg/kg 96406 (27,949–224,426) copies per mL). There may be a threshold concentration of miR-122 in the circulation that needs to be reached before there is an increase in the kidney. Fig. 2 in our paper also supports this possibility. In Fig. 2c there was a small increase in miR-122 in the serum of null mice treated with the 150 mg/kg dose of paracetamol which did not result in an increase in the kidney (Fig. 2e and f). Transfer to the kidney was also demonstrated using a model of ischemic injury to the myocardium with miR-499 as the target microRNA. This demonstrates that the kidney is a key node that internalises microRNA released from injured organs. Recent *in vivo* data demonstrate that miR-122 released from the injured liver can induce acute lung inflammation by activating alveolar macrophages [26]. In our studies there was not a significant decrease in lung miR-122 following liver microRNA depletion. In contrast to the kidney, this suggests that microRNA transfer from the liver to the lung is not significant in the healthy uninjured state. In humans, the cargo of miR-122 in urinary ECVs is substantially increased when the liver is injured which supports our mouse model of liver to kidney transfer occurring in human disease. The mechanism by which miR-122 goes from the circulation to urinary ECVs remains to be defined. It could be that ECVs transfer from blood to urine. Alternatively, miR-122 containing ECVs could be generated *de novo* by the kidney tubular cells when the liver is injured. However, our mouse model clearly demonstrates that the increase in miR-122 in the kidney cells originates from the injured liver. Therefore, we postulate that the increase in urinary ECV miR-122 supports the existence of liver to kidney transfer in humans.

There are many different pathways that could be regulated in the kidney by microRNA originating from other organs and this will be a subject of further research. We focussed on CYP2E1 because it is established as being regulated by miR-122 and it is a key enzyme in the drug metabolism that underlies liver and kidney toxicity. Our data demonstrate that CYP2E1 RNA and protein expression and enzymatic activity is regulated by liver-derived microRNA in the liver and the kidney. As others have reported, the consequence of microRNA depletion in the liver was increased injury following paracetamol exposure [13,14]. Interestingly, in the liver, miR-122 was substantially increased in the hepatocytes surrounding the necrotic core (miR-122 expression was lost from the core). This effect was not apparent when bulk tissue expression was measured by PCR. Theoretically, transfer of miR-122 may reduce drug toxicity in the kidney by down-regulating CYP enzyme activity. Paracetamol is not a robust model for acute kidney injury with multiple studies demonstrating little or no injury occurs despite significant liver injury [24]. Future *in vivo* studies could explore the effect of hepatic microRNA depletion on the renal response in established rodent kidney injury models such as ischaemia/reperfusion, cisplatin nephrotoxicity or sepsis models. To start this process of exploring the potential role of hepatic-renal microRNA signalling we demonstrated that ECVs from mice with liver injury prevented cisplatin toxicity in primary cells, which is reported to be dependent on CYP2E1 activity. John *et al.* reported that patients with acute liver failure who die have lower circulating miR-122, which is consistent with miR-122 inter-organ transfer having a possible protective effect in humans [27].

In summary, there is physiological transfer of microRNA from the liver to the kidneys that is increased by hepatotoxicity and regulates kidney CYP enzymes. miR-122 represents the majority of the hepatocyte microRNA cargo and, following hepatotoxicity in humans, it is the highest concentration microRNA in the circulation. We propose miR-122 release (and potentially other microRNA species) has the potential to mediate resistance to drug toxicity in the kidney and may be, therefore, acting as a break on multi-organ failure in the context of liver injury. With further research and development, miR-122 may represent a treatment to protect renal function in patients. Although there is a large endogenous release of miR-122 into the circulation with liver injury, it has a short half-life compared to ALT. We speculate that continued treatment with miR-122 in patients with significant liver injury could offer renal protection.

## 5 Contributions

All authors read and approved the final version of the manuscript. Experiments were performed by OM, EM, JT and PSL. Technical support was provided by IT, AS, RS, HR, KM. Histological analysis by TJK. Supervision was performed by GAG, CG, KP, LD, ND, MB NH and DW. The work was directed by JD.

## Data sharing statement

Data are available on request for corresponding author.

## Declaration of Competing Interest

Author JWD is a member of the expert advisory group for the EU IMI funded TransBioLine Consortium. Author LD supervises a PhD studentship co-funded by Regulus Therapeutics and GSK.

## Appendix Supplementary materials

Supplementary Figure 1. a-i Liver histology from untreated Dicer^flox/flox^ mice (A) and mice treated with AAV8-null or Cre with tissue collected 1-4 weeks after treatment (B-I). Higher power liver histology images from mice with elevated ALT (j-m). Serum ALT was increased by AAV8 without a difference between Cre and null (n). Data are represented individual mice with bars representing median and IQR. Image, image 1

Supplementary Figure 2. a The fold change over time in organ Dicer mRNA expression after treatment of Dicer^flox/flox^ mice with AAV8 vector expressing or not expressing Cre Recombinase (Cre). For each organ the expression of Dicer is expressed as a percentage of the untreated Dicer^flox/flox^ mice (baseline). Data are normalised to 18S. Filled symbols represent mice receiving Cre-AAV8. Unfilled symbols represent Null-AAV8. Symbols represent the time point median and the error bars define the inter-quartile range. N=20 for each AVV8/microRNA combination (N=5 for each time point). * = P<0.05 compared with baseline by Mann-Whitney Test. b Western blot of Dicer protein expression in liver and kidney. A = baseline untreated mouse. B, D, F, H AAV8 null treated mice and tissue collected 1, 2, 3 and 4 weeks later. C, E, G, I AAV8 Cre treated mice and tissue collected 1, 2, 3 and 4 weeks later. Image, image 2

Supplementary Figure 3. Full western blots for DICER. Red arrow indicates expected molecular weight. Image, image 3

Supplementary Figure 4. Absolute expression of miR-122 in the liver, kidney cortex and medulla after treatment of Dicer^flox/flox^ mice with AAV8 vector expressing or not expressing Cre Recombinase (Cre). miR-122 is normalized by 3 different housekeeping genes, SNORD68, SNORD95 and U6. Data are presented as 2ΔCt values. Statistical significance was determined by Mann-Whitney Test. * = P=0.02 * * = P0.008 compared to week 0 (untreated mice). Data are represented as Tukey plots. N=5 per time point group. Image, image 4

Supplementary Figure 5. Liver injury due to paracetamol induces a change in miR-122 expression which is absent following conditional knock out of Dicer. DICER ^flox/flox^ mice were treated with AAV8 vector expressing or not expressing Cre Recombinase (Cre or Null). 3 weeks after AAV8 treatment mice received paracetamol 150 or 300 mg/kg (or vehicle (0)). In situ hybridisation for miR-122 in the liver was performed as described in methods. U6 and scrambled microRNA probe controls are also presented. Image, image 5

Supplementary Figure 6. Dicer^flox/flox^ mice were treated with AAV8 vector expressing or not expressing Cre Recombinase (Cre or null). 3 weeks after AAV8 treatment mice received paracetamol 150 or 300 mg/kg (or vehicle (0)), then liver, kidney cortex and kidney medulla were harvested 6 hours later. Baseline = Dicer^flox/flox^ mice not receiving AAV8. Serum alanine transaminase activity (ALT) (a) and liver necrosis scores are presented in graphs (b). Liver necrosis scored as per methods. N=5 per group. Statistical significance was determined by Mann-Whitney Test. Data are represented as Tukey plots. Representative liver histology from AAV8-null and Cre treated Dicer^flox/flox^ mice are presented with vehicle or paracetamol treatment (c). Image, image 6

Supplementary Figure 7. Dicer^flox/flox^ mice were treated with AAV8 vector expressing or not expressing Cre Recombinase (Cre or Null). 3 weeks after AAV8 treatment mice received paracetamol 150 or 300 mg/kg (or vehicle (0)) ip, then liver, kidney cortex and kidney medulla were harvested 6 hours later. Untreated = Dicer^flox/flox^ mice not receiving AAV8. Dicer mRNA expression is expressed as GAPDH(Ct) - DICER (Ct). N=5 per group. Statistical significance was determined by Mann-Whitney Test. Data are represented individual mice with bars representing median and IQR (N=5 per group). Image, image 7

Supplementary Figure 8. Six hours after treatment with paracetamol (300mg/kg ip) kidney cells were FACS sorted and miR-122 was measured by PCR. A standard curve was used to calculate the absolute copy number per cell. Statistical significance was determined by Mann-Whitney Test. Data are represented as Tukey plots. N=5 per group. Image, image 8

Supplementary Figure 9. Myocardial injury was induced in mice by coronary artery ligation (CAL). After 2 or 6 hours tissue and serum was collected. Sham operated mice had thoracotomy performed but no ligation. In the left ventricle and kidney cortex miR-499 expression is expressed as U6(ct) - miR (ct). In serum miR-499 is normalised by spike in miR-39. Statistical significance was determined by Mann-Whitney Test. Data are represented individual mice with bars representing median and IQR. N=5 per group. Image, image 9

Supplementary Figure 10. Full western blot for CYP2E1 in kidney (see figure 5). Image, image 10

Supplementary Figure 11. a-d. Bright-field micrographs of primary renal cell preparations in culture. Cells isolated from Percoll fractions were grown in culture medium for 48 hours. Bright-field microscopy of cell monolayers from F1 (a), F2 (b), F3 (c) and F4 (d). Each image is representative of 3 samples from each fraction. e) Cultured primary renal cells retain alkaline phosphatase activity. Alkaline phosphatase activity was retained in cultured cells, isolated from Percoll bands F1-4. Cultured cells within F4 had significantly higher alkaline phosphatase activity than the 3 other bands, when normalized to total protein. Data are shown as Tukey boxplots, n=4. Statistical significance was determined by one- way AVOVA with Tukey's multiple comparison test. f) Western blots to estimate the abundance of NHE3 in primary renal cells. Cells were isolated from each Percoll fraction and homogenized. 12 μg of protein was loaded per lane. NHE3 band detected at ∼84 kDa g). Confocal image of F4 cell monolayer confirms expression of archetypal proximal tubule proteins. Image shows KIM-1 (green), nuclei stained with DAPI (blue) and actin within the cell membrane (red). Images are representative of 3 samples. Scale bars are 20 μm. Image, image 11

Image, application 12

Image, application 13
