# Supplementary figures and images for: Transfer of hepatocellular microRNA regulates cytochrome P450 2E1 in renal tubular cells
Source: eBioMedicine. 2020 Nov 21;62:103092. doi: 10.1016/j.ebiom.2020.103092 (PMC7689533; doi:10.1016/j.ebiom.2020.103092)

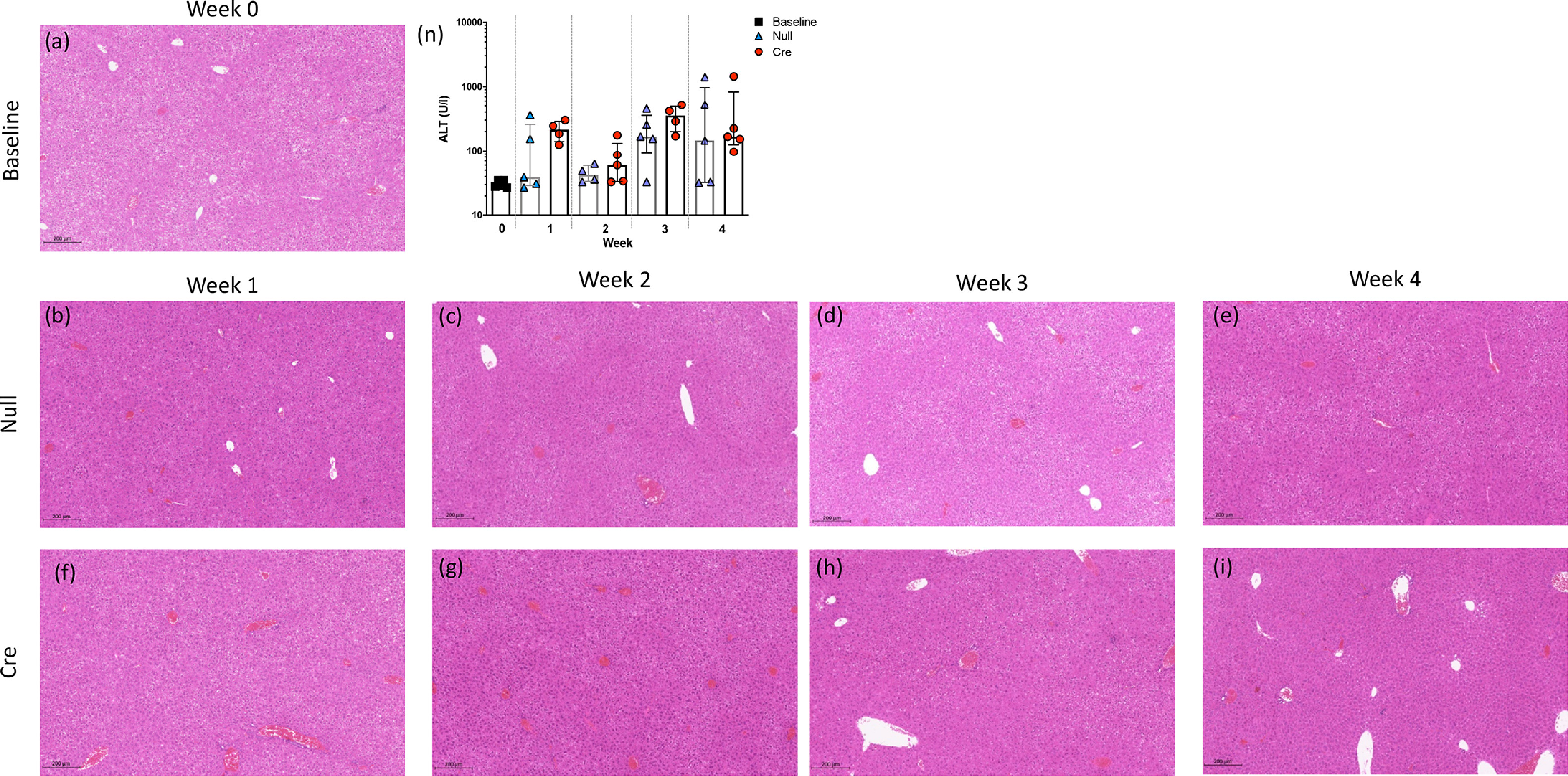

Supplement: Supplementary file 1 — Supplementary Figure 1. a-i Liver histology from untreated Dicerflox/flox mice (A) and mice treated with AAV8-null or Cre with tissue collected 1-4 weeks after treatment (B-I). Higher power liver histology images from mice with elevated ALT (j-m). Serum ALT was increased by AAV8 without a difference between Cre and null (n). Data are represented individual mice with bars representing median and IQR. [file mmc1.jpg]

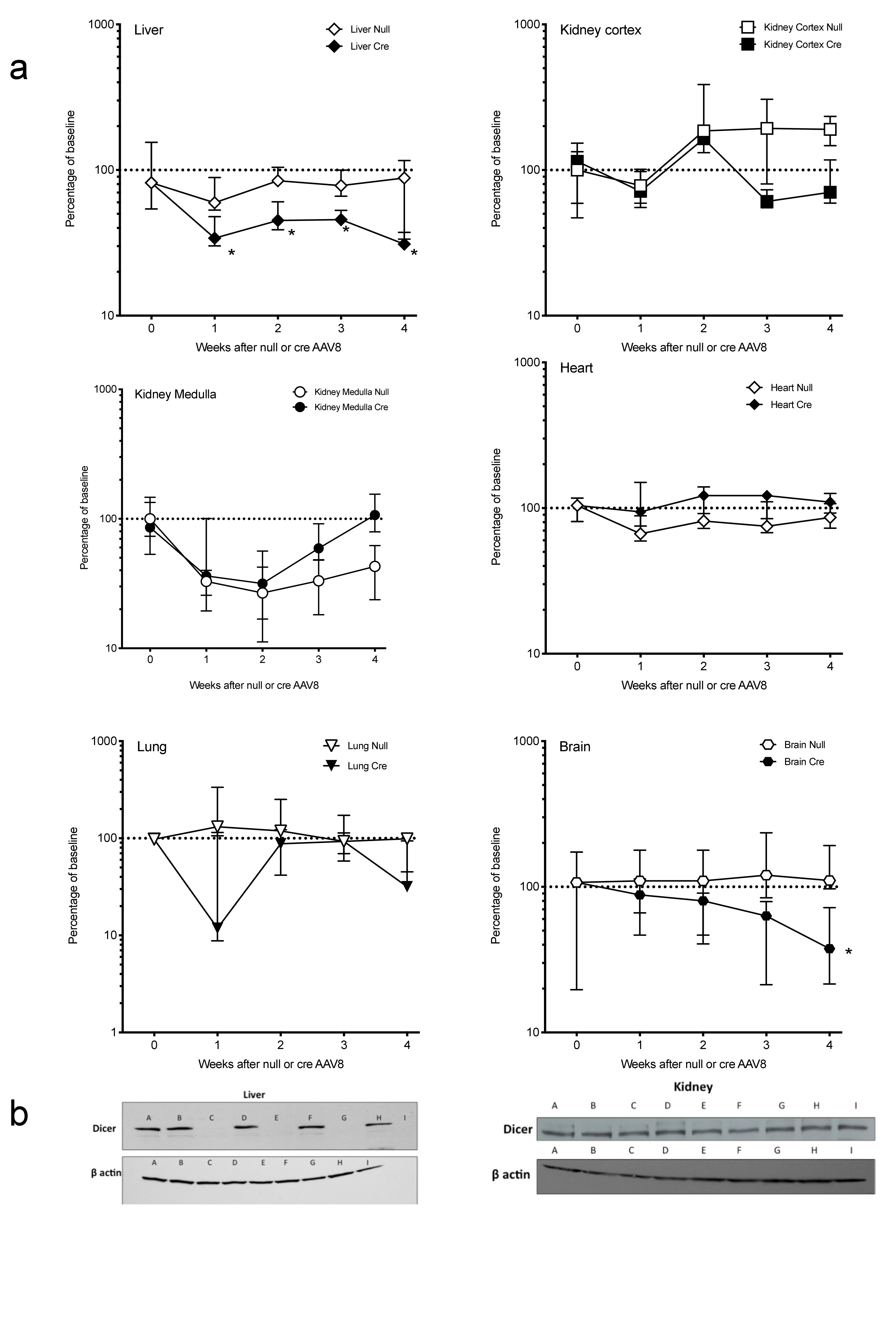

Supplement: Supplementary file 2 — Supplementary Figure 2. a The fold change over time in organ Dicer mRNA expression after treatment of Dicerflox/flox mice with AAV8 vector expressing or not expressing Cre Recombinase (Cre). For each organ the expression of Dicer is expressed as a percentage of the untreated Dicerflox/flox mice (baseline). Data are normalised to 18S. Filled symbols represent mice receiving Cre-AAV8. Unfilled symbols represent Null-AAV8. Symbols represent the time point median and the error bars define the inter-quartile range. N=20 for each AVV8/microRNA combination (N=5 for each time point). * = P<0.05 compared with baseline by Mann-Whitney Test. b Western blot of Dicer protein expression in liver and kidney. A = baseline untreated mouse. B, D, F, H AAV8 null treated mice and tissue collected 1, 2, 3 and 4 weeks later. C, E, G, I AAV8 Cre treated mice and tissue collected 1, 2, 3 and 4 weeks later. [file mmc2.jpg]

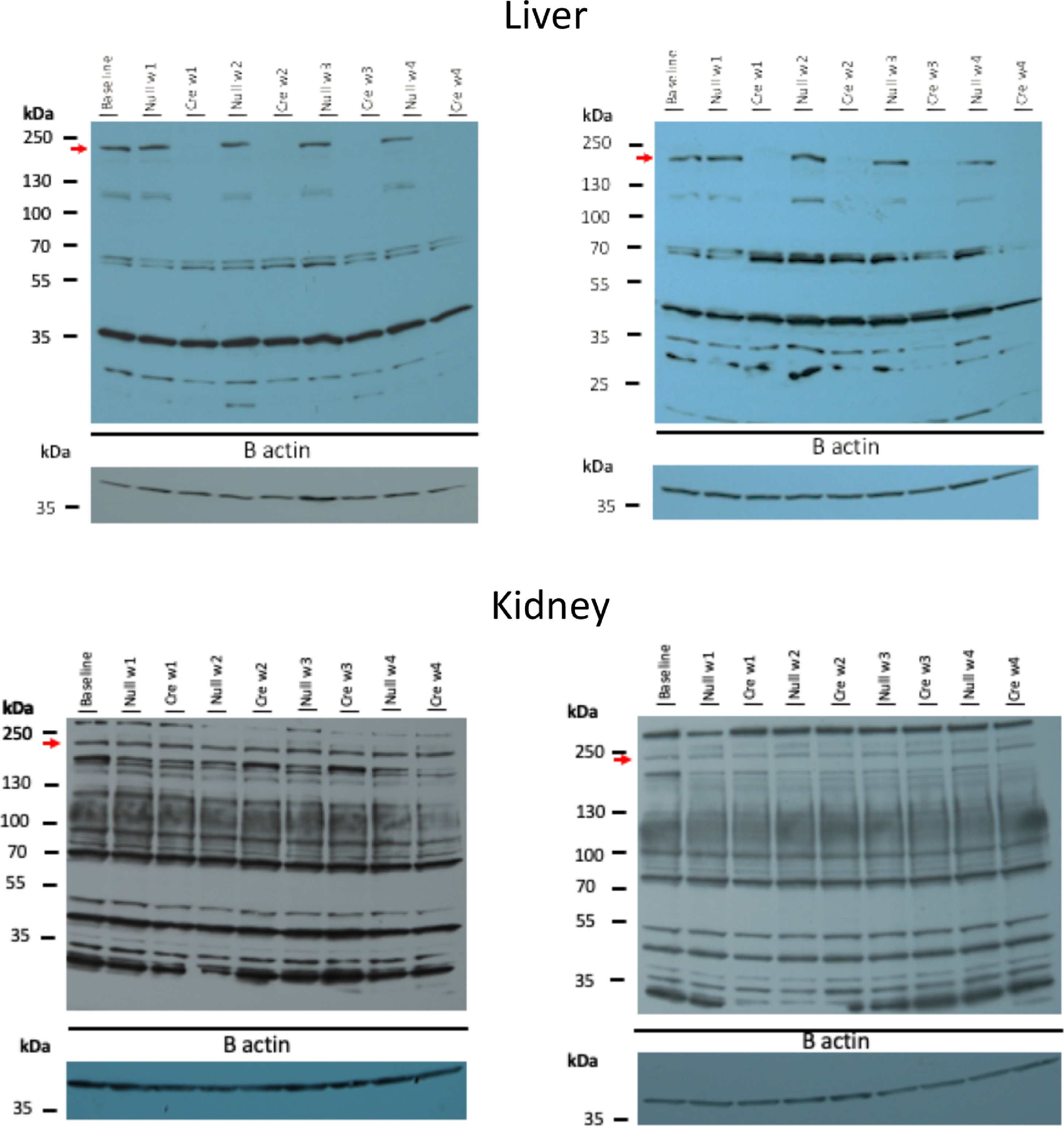

Supplement: Supplementary file 3 — Supplementary Figure 3. Full western blots for DICER. Red arrow indicates expected molecular weight. [file mmc3.jpg]

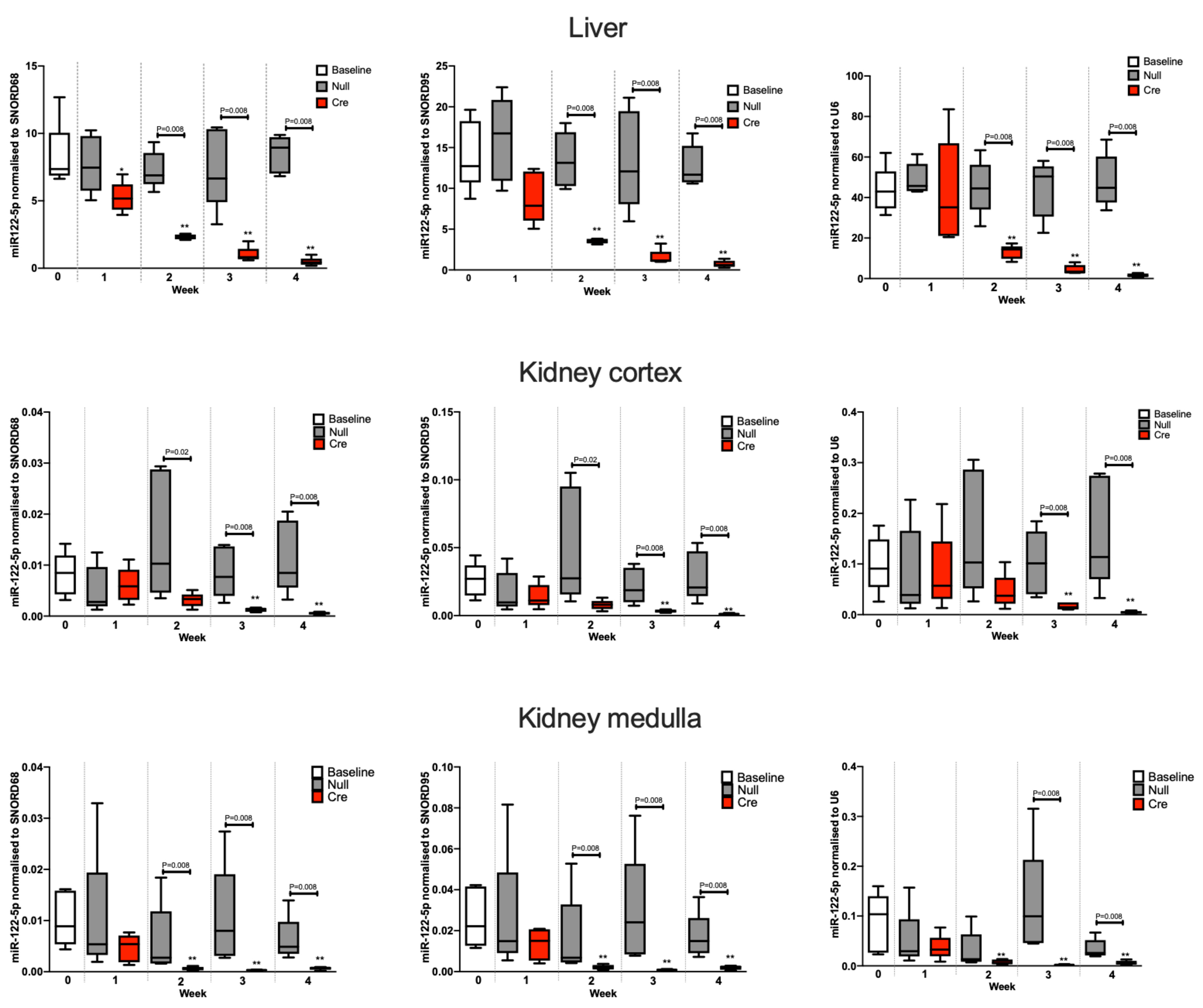

Supplement: Supplementary file 4 — Supplementary Figure 4. Absolute expression of miR-122 in the liver, kidney cortex and medulla after treatment of Dicerflox/flox mice with AAV8 vector expressing or not expressing Cre Recombinase (Cre). miR-122 is normalized by 3 different housekeeping genes, SNORD68, SNORD95 and U6. Data are presented as 2ΔCt values. Statistical significance was determined by Mann-Whitney Test. * = P=0.02 * * = P0.008 compared to week 0 (untreated mice). Data are represented as Tukey plots. N=5 per time point group. [file mmc4.jpg]

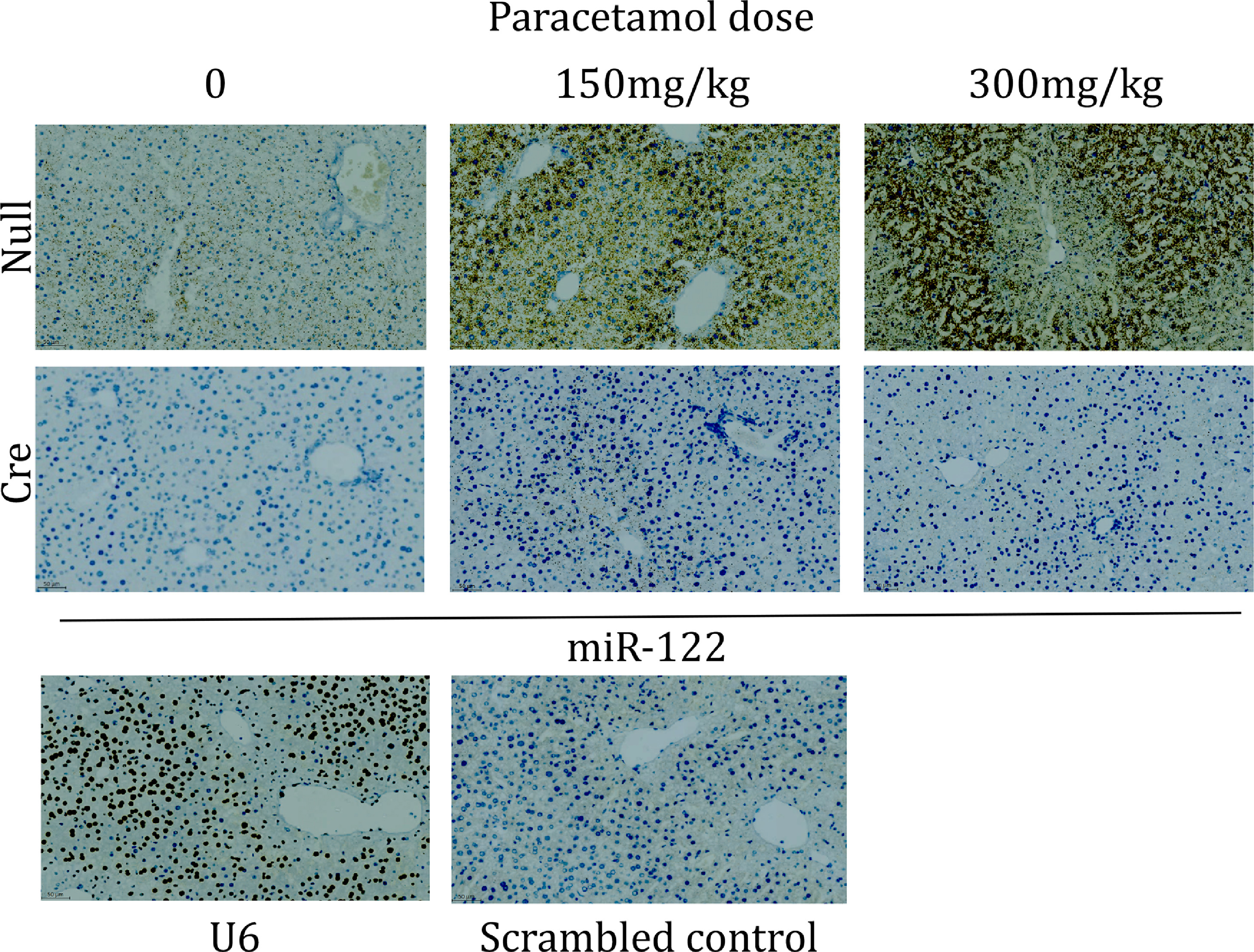

Supplement: Supplementary file 5 — Supplementary Figure 5. Liver injury due to paracetamol induces a change in miR-122 expression which is absent following conditional knock out of Dicer. DICER flox/flox mice were treated with AAV8 vector expressing or not expressing Cre Recombinase (Cre or Null). 3 weeks after AAV8 treatment mice received paracetamol 150 or 300 mg/kg (or vehicle (0)). In situ hybridisation for miR-122 in the liver was performed as described in methods. U6 and scrambled microRNA probe controls are also presented. [file mmc5.jpg]

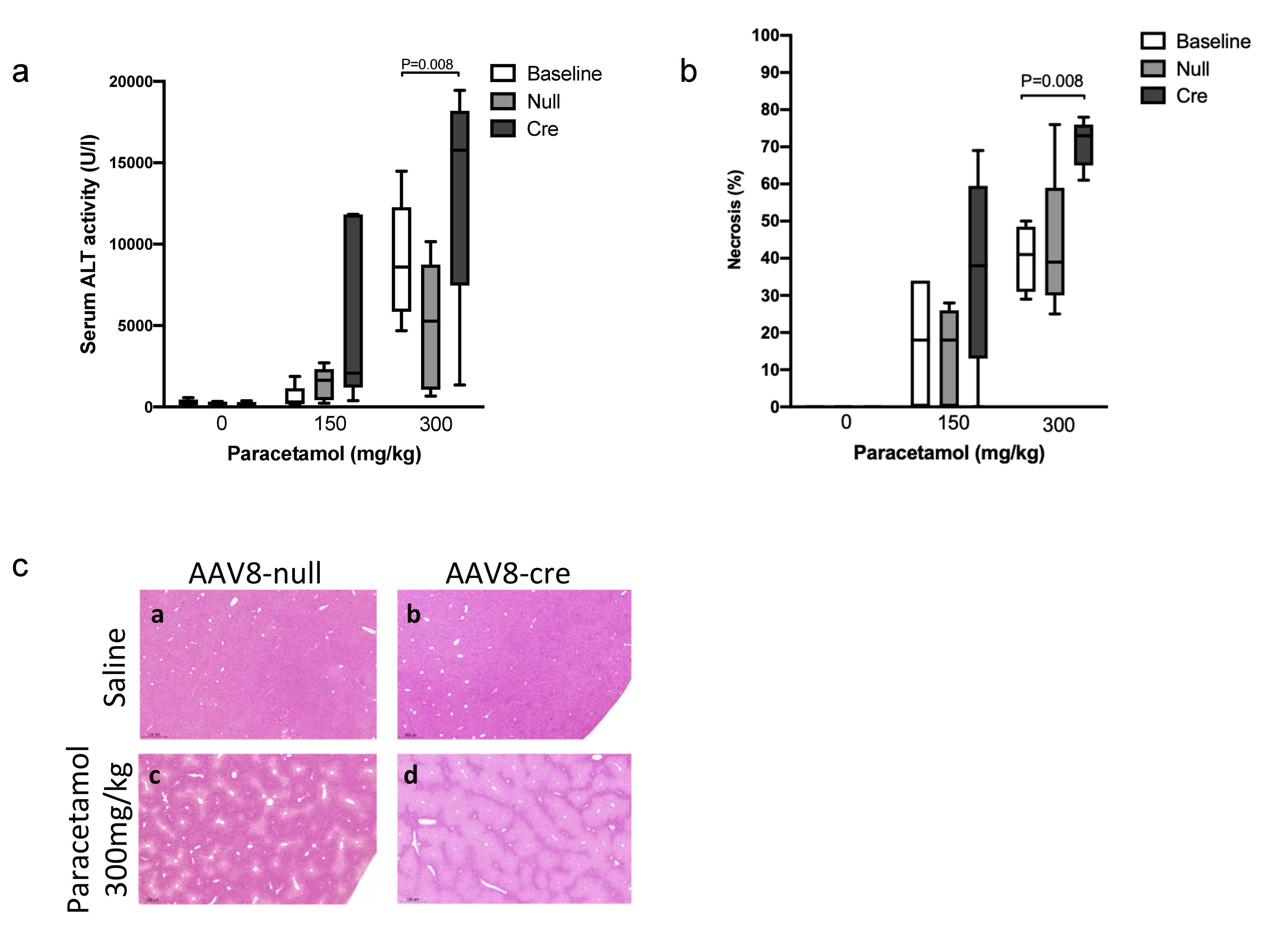

Supplement: Supplementary file 6 — Supplementary Figure 6. Dicerflox/flox mice were treated with AAV8 vector expressing or not expressing Cre Recombinase (Cre or null). 3 weeks after AAV8 treatment mice received paracetamol 150 or 300 mg/kg (or vehicle (0)), then liver, kidney cortex and kidney medulla were harvested 6 hours later. Baseline = Dicerflox/flox mice not receiving AAV8. Serum alanine transaminase activity (ALT) (a) and liver necrosis scores are presented in graphs (b). Liver necrosis scored as per methods. N=5 per group. Statistical significance was determined by Mann-Whitney Test. Data are represented as Tukey plots. Representative liver histology from AAV8-null and Cre treated Dicerflox/flox mice are presented with vehicle or paracetamol treatment (c). [file mmc6.jpg]

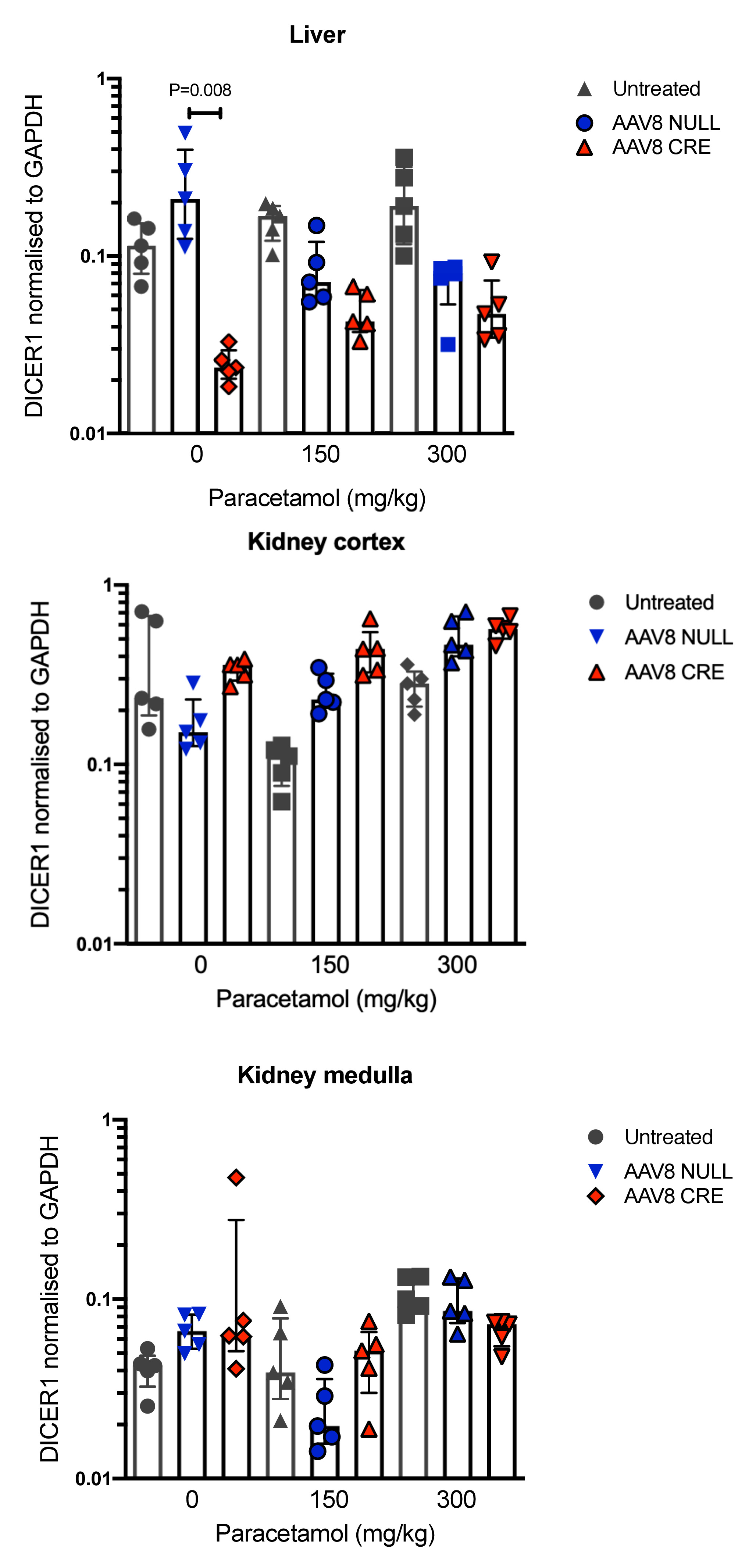

Supplement: Supplementary file 7 — Supplementary Figure 7. Dicerflox/flox mice were treated with AAV8 vector expressing or not expressing Cre Recombinase (Cre or Null). 3 weeks after AAV8 treatment mice received paracetamol 150 or 300 mg/kg (or vehicle (0)) ip, then liver, kidney cortex and kidney medulla were harvested 6 hours later. Untreated = Dicerflox/flox mice not receiving AAV8. Dicer mRNA expression is expressed as GAPDH(Ct) - DICER (Ct). N=5 per group. Statistical significance was determined by Mann-Whitney Test. Data are represented individual mice with bars representing median and IQR (N=5 per group). [file mmc7.jpg]

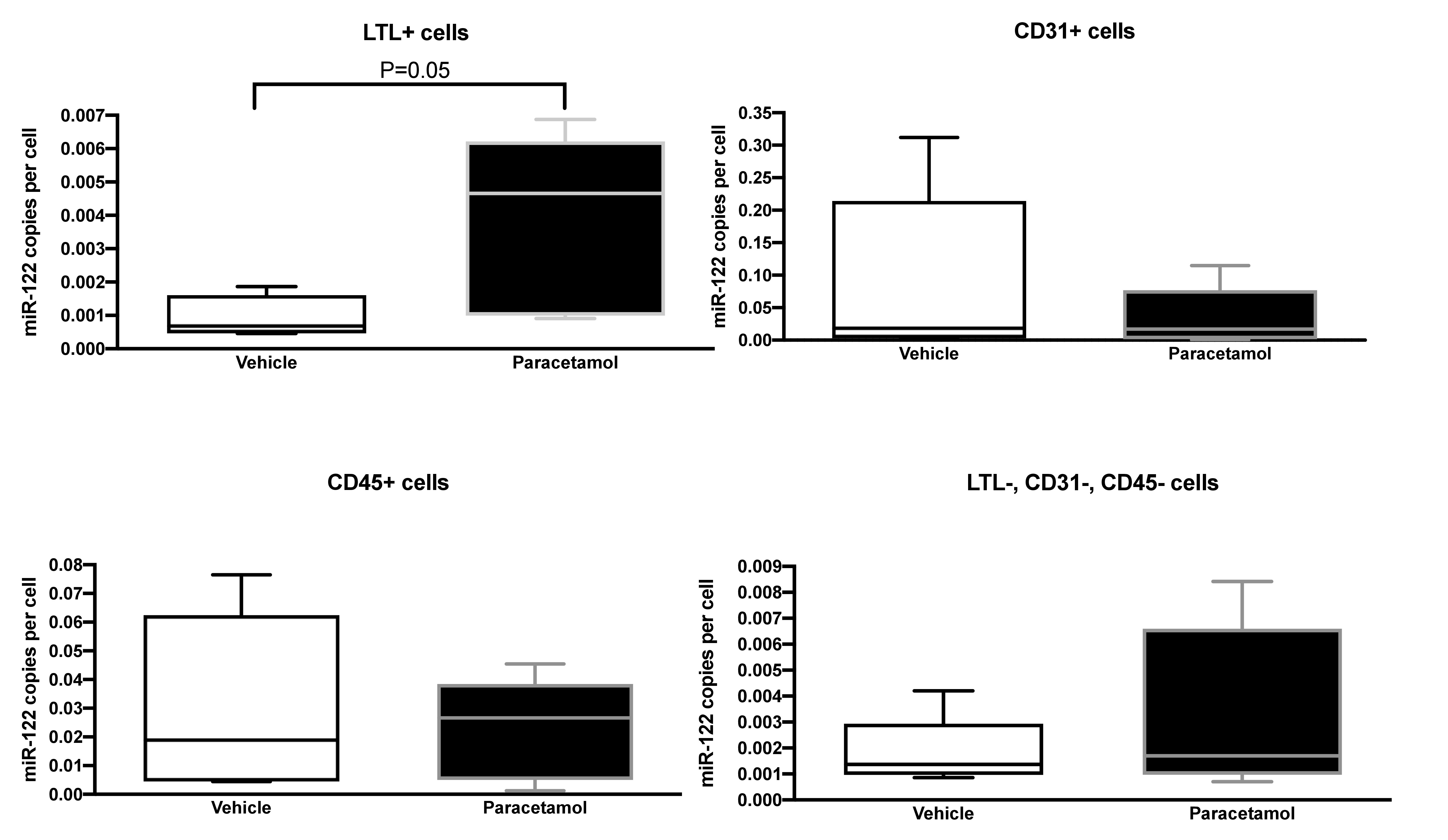

Supplement: Supplementary file 8 — Supplementary Figure 8. Six hours after treatment with paracetamol (300mg/kg ip) kidney cells were FACS sorted and miR-122 was measured by PCR. A standard curve was used to calculate the absolute copy number per cell. Statistical significance was determined by Mann-Whitney Test. Data are represented as Tukey plots. N=5 per group. [file mmc8.jpg]

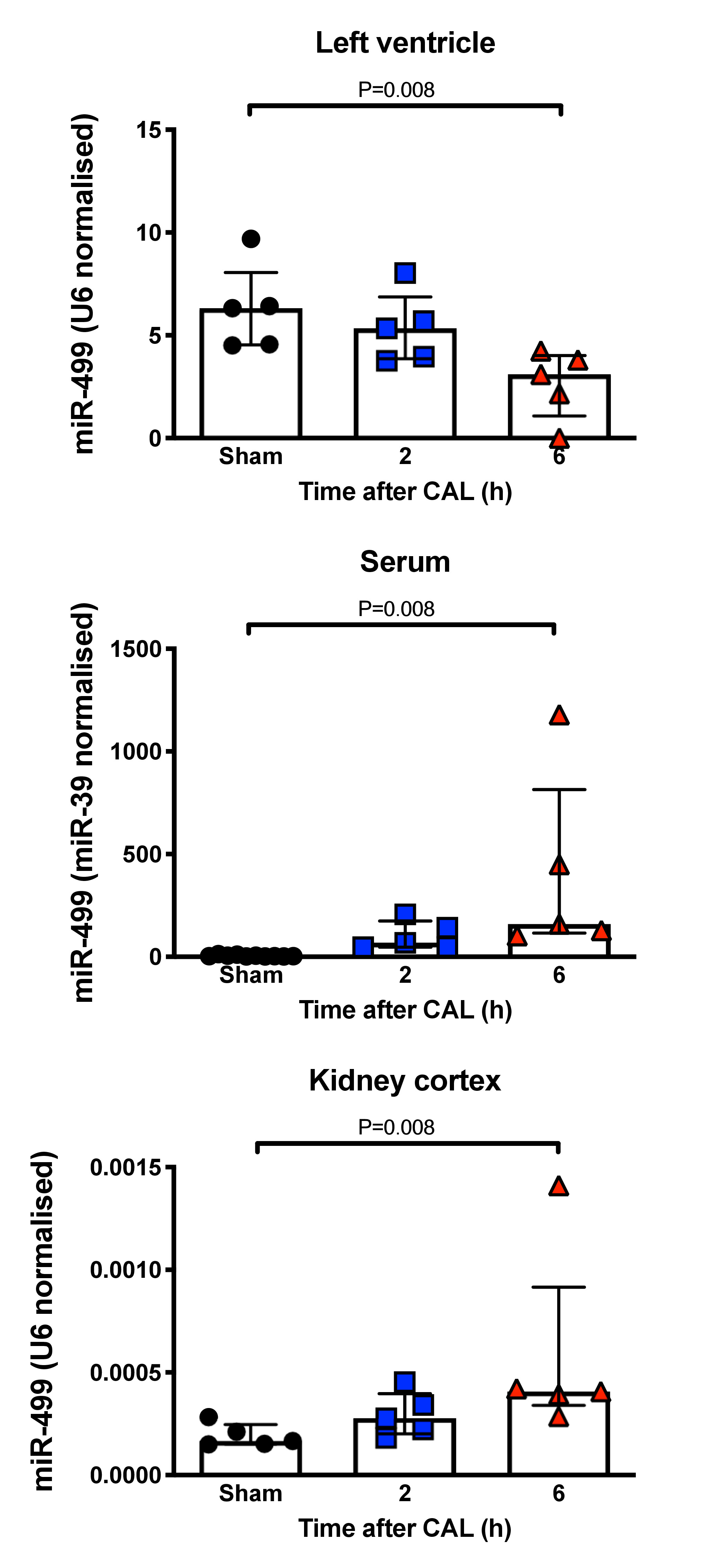

Supplement: Supplementary file 9 — Supplementary Figure 9. Myocardial injury was induced in mice by coronary artery ligation (CAL). After 2 or 6 hours tissue and serum was collected. Sham operated mice had thoracotomy performed but no ligation. In the left ventricle and kidney cortex miR-499 expression is expressed as U6(ct) - miR (ct). In serum miR-499 is normalised by spike in miR-39. Statistical significance was determined by Mann-Whitney Test. Data are represented individual mice with bars representing median and IQR. N=5 per group. [file mmc9.jpg]

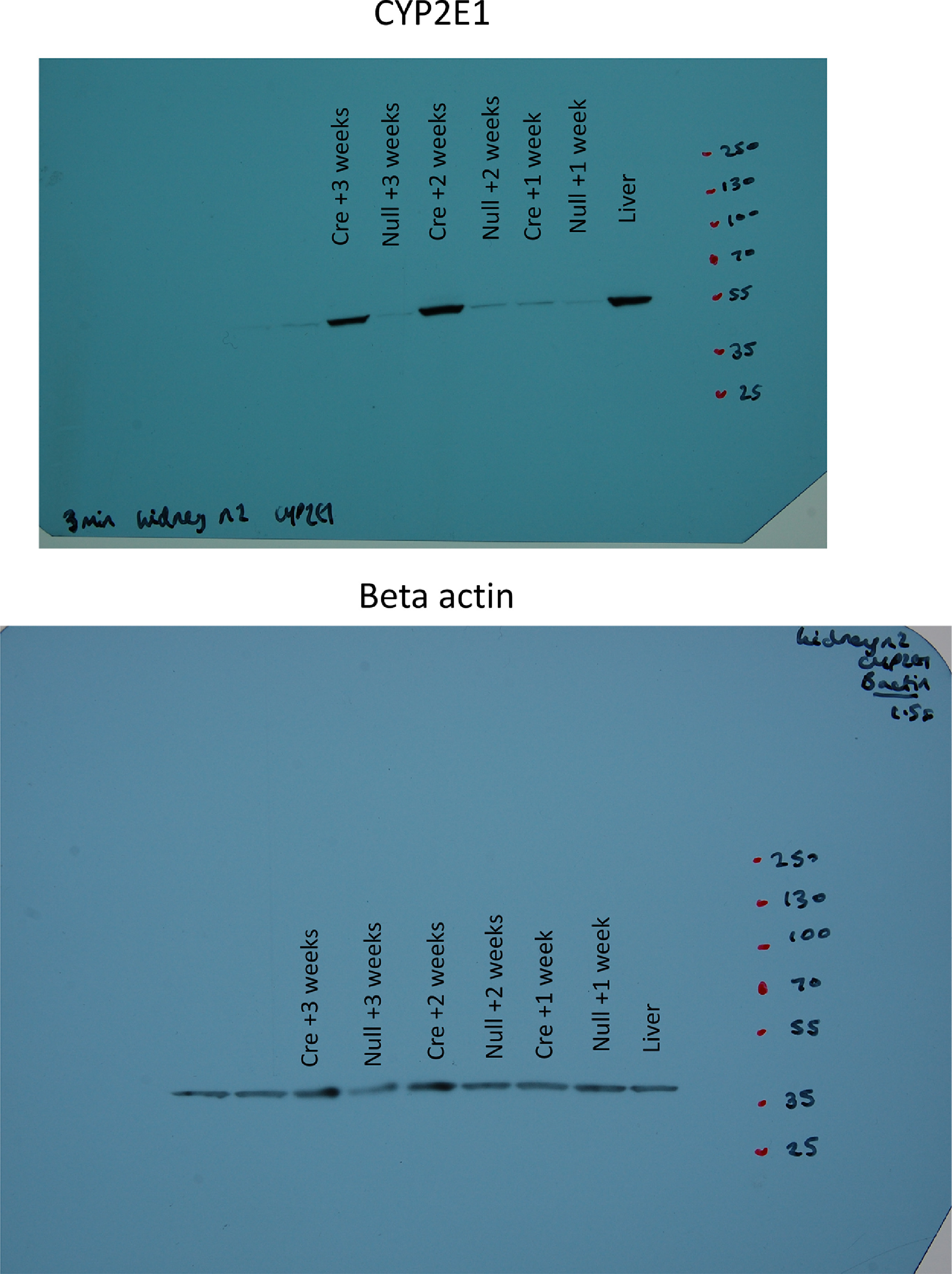

Supplement: Supplementary file 10 — Supplementary Figure 10. Full western blot for CYP2E1 in kidney (see figure 5). [file mmc10.jpg]

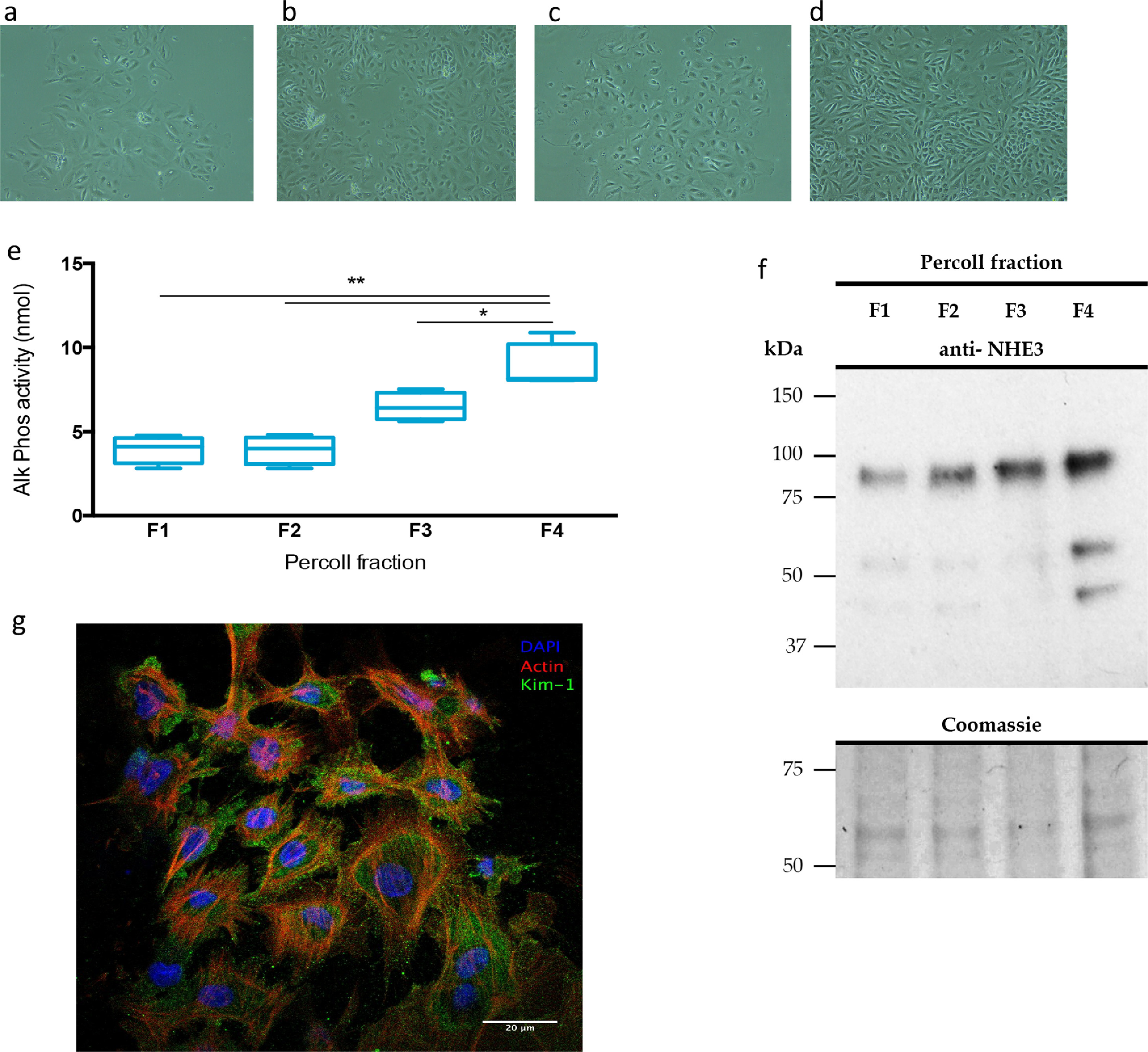

Supplement: Supplementary file 11 — Supplementary Figure 11. a-d. Bright-field micrographs of primary renal cell preparations in culture. Cells isolated from Percoll fractions were grown in culture medium for 48 hours. Bright-field microscopy of cell monolayers from F1 (a), F2 (b), F3 (c) and F4 (d). Each image is representative of 3 samples from each fraction. e) Cultured primary renal cells retain alkaline phosphatase activity. Alkaline phosphatase activity was retained in cultured cells, isolated from Percoll bands F1-4. Cultured cells within F4 had significantly higher alkaline phosphatase activity than the 3 other bands, when normalized to total protein. Data are shown as Tukey boxplots, n=4. Statistical significance was determined by one- way AVOVA with Tukey's multiple comparison test. f) Western blots to estimate the abundance of NHE3 in primary renal cells. Cells were isolated from each Percoll fraction and homogenized. 12 μg of protein was loaded per lane. NHE3 band detected at ∼84 kDa g). Confocal image of F4 cell monolayer confirms expression of archetypal proximal tubule proteins. Image shows KIM-1 (green), nuclei stained with DAPI (blue) and actin within the cell membrane (red). Images are representative of 3 samples. Scale bars are 20 μm. [file mmc11.jpg]
