# Supplementary material for: Transfer of hepatocellular microRNA regulates cytochrome P450 2E1 in renal tubular cells
Source: eBioMedicine. 2020 Nov 21;62:103092. doi: 10.1016/j.ebiom.2020.103092 (PMC7689533; doi:10.1016/j.ebiom.2020.103092)
Supplement: Supplementary file 12 [file mmc12.docx]

| **Target (immunogen)** | **Host and Conjugate** | **Source and Catalogue no.** | **Dilution factor** |
| --- | --- | --- | --- |
| **Primary antibodies** | | | |
| Dicer | Rabbit | Sigma Aldrich, SAB004200087 | 1:200 |
| Cytochrome P450 2E1 | Rabbit | Abcam, ab28146 | 1:5000 |
| β-actin | Mouse | Sigma Aldrich, A2228 | 1:20,000 |
| **Secondary antibodies** | | | |
| Rabbit IgG | Goat, HRP | Vector Laboratories,  PI-1000-1 | 1:3000 |
| Mouse IgG | Horse, HRP | Vector Laboratories,  PI-2000-1 | 1:3000 |

Supplementary Table 1. Antibodies used in Western Blot experiments

| **Group** | **Age (yrs)** | **% Female** | **ALT (U/L)** | **PT (sec)** | **Creatinine (umol/L)** | **% Died or had liver transplant** |
| --- | --- | --- | --- | --- | --- | --- |
| No liver injury | 45  (19-52) | 75 | 36  (13-102) | 13  (12-15) | 75  (60-84) | 0 |
| Acute liver injury | 44  (35-50) | 64 | 5699  (3885-8712) | 81  (54-96) | 298  (91-447) | 36 |

Supplementary Table 2. Characteristics of the patient groups who were grouped by either having no liver injury or acute liver injury (ALT>1000 U/L) after paracetamol overdose. Continuous data are presented as the median (IQR). ALT = alanine transaminase. PT= prothrombin time.
